# Evaluation of an SMS-based mHealth intervention to enhance early infant diagnosis follow-up testing and assessment of postnatal prophylaxis

**DOI:** 10.4102/sajhivmed.v22i1.1301

**Published:** 2021-11-24

**Authors:** Anele Dube-Pule, Brian C. Zanoni, Cathy Connolly, Majahonkhe Shabangu, Moherndran Archary

**Affiliations:** 1 Department of Paediatrics and Child Health, Faculty of Medicine, University of KwaZulu-Natal, Durban, South Africa; 2 Department of Pediatrics, Division of Infectious Diseases, Emory University School of Medicine, Atlanta, United States of America; 3 School of Public Health, University of KwaZulu-Natal, Durban, South Africa; 4 Sawubona Health Inc., Malden, Massachusetts, United States of America; 5 Department of Human Biology, Division of Biomedical Engineering, University of Cape Town, Cape Town, South Africa; 6 Department of Paediatrics and Child Health, College of Health Sciences, University of KwaZulu-Natal, Durban, South Africa; 7 Department of Paediatrics, King Edward VIII Hospital, Durban, South Africa

**Keywords:** mHealth, early infant diagnosis, HIV DNA PCR, infant prophylaxis, high-risk mothers, low-risk mothers, SMS reminders, PMTCT

## Abstract

**Background:**

Adherence to infant antiretroviral (ARV) postnatal prophylaxis and early infant diagnosis (EID) uptake is low in Africa. Promoting EID and adherence are necessary for this age group.

**Objectives:**

We evaluated an SMS-based mobile health (mHealth) intervention to enhance adherence to ARV prophylaxis and knowledge of EID and prevention of mother-to-child transmission (PMTCT) among high-risk and low-risk mother–infant pairs.

**Method:**

Two hundred and fifty-one mothers were recruited from King Edward VIII Hospital between December 2018 and October 2019. Participant information was captured, and SMS reminders were sent postnatally to promote immunisation attendance. Follow-up HIV polymerase chain reaction (PCR) test results were reviewed, and telephonic interviews were utilised for qualitative data.

**Results:**

In all, 73.3% of infants had HIV PCR tests performed at 10 weeks. This high rate could be attributed to the mHealth intervention as this is considerably higher than other national studies, though not statistically significant compared to rates reported in the district at the same time. Factors that have impacted follow-up EID rates include poor maternal knowledge of EID time points and inadequate implementation of national PMTCT protocols. High-risk mothers were younger, commenced antenatal clinic visit later, were less knowledgeable on prophylaxis and have lower-birthweight infants than lower-risk mothers.

**Conclusion:**

mHealth can play an important role in improving EID by increasing maternal knowledge. Further studies should focus on whether maternal education over an mHealth platform can increase knowledge on PMTCT and subsequently increase EID.

## Introduction

Mother-to-child transmission (MTCT) rates for HIV declined impressively by 84% between 2008 and 2015 in South Africa (SA); however, since then, there has been a slower decline, jeopardising the World Health Organization (WHO) targets of preventing 6 million new infections among children by 2030.^1^ Despite high antenatal HIV testing rates and universal maternal antiretroviral treatment (ART), SA has yet to eliminate MTCT, as the infant HIV transmission rate at 18 months in 2019 was still 3%.^2^ Gaps in the prevention of mother-to-child transmission (PMTCT) cascade after delivery contribute to these ongoing HIV transmissions. These include poor monitoring of HIV-negative mothers until the end of breastfeeding, poor viral load (VL) monitoring among HIV-positive mothers, poor adherence to both maternal ART and infant antiretroviral (ARV) prophylaxis and low rates of repeat testing for early infant diagnosis (EID) at the recommended 10-week, 6-month and 18-month visits.^3,4,5^

Efforts to decrease postnatal MTCT rates should be directed at maintaining a suppressed maternal VL through early ART initiation and ART adherence, together with adherence to infant ARV postnatal prophylaxis (PNP). High rates of poor adherence to maternal ART and PNP have been reported in Africa: infant PNP adherence rates range between 72.0% and 85.0% during the 6 weeks of nevirapine prophylaxis,^5,6,7^ while in 2019 maternal ART adherence was reported at only 63.4%.^5^

Early infant diagnosis is vital in promoting early ART initiation for infants diagnosed with HIV. The Children with HIV early antiretroviral therapy (CHER) trial demonstrated a 76% and 75% reduction in morbidity and mortality, respectively, for infants living with HIV who initiated ART before three months of age, making early diagnosis and early ART initiation critical.^8^ The SA EID guidelines changed in line with WHO recommendations in 2015 to include an HIV polymerase chain reaction (PCR) test at birth and 10 weeks for all HIV-exposed infants. In addition, high-risk infants were also required to have an 18-week HIV PCR. This has subsequently been changed to a 6-month HIV PCR for all exposed infants.^9^ In addition, all HIV-exposed infants are required to be tested at six weeks post-cessation of breastfeeding and, lastly, to have an HIV antibody test at the age of 18 months, which, if positive, should be followed with an HIV PCR.^10^ While EID coverage at birth is generally high, 89% in 2018,^2^ largely as a result of the high percentage of deliveries within healthcare facilities in SA,^10^ follow-up EID testing at other time points is low.^3,11^ Causes of the low uptake of follow-up EID testing in sub-Saharan Africa (SSA) include low rates of retention in care in PMTCT services,^12,13^ poor maternal knowledge about the vertical transmission of HIV,^14^ fear of maternal stigmatisation and lack of privacy at follow-up clinics.^15^ In addition, only 35% of mothers living with HIV reported intentions to self-request for infant testing at immunisation visits.^15^

Several studies have reported the successful use of mobile devices such as phones to support healthcare practice (mHealth), mostly to improve appointment attendance and adherence to medication.^16,17,18,19^ Following the WHO recommendation to use SMS programmes to support ART, several mHealth studies to improve adherence have included people living with HIV as well as EID.^16,17,18,19^ Other positive benefits of mHealth include educating clients, encouraging behaviour change, enhancing decision-making and enabling communication between care providers. mHealth is inexpensive and easy to implement.^20^ The use of mHealth interventions has been understudied in SA despite a 150% saturation of mobile phones.^21^

MomConnect, a gestation-specific interactive SMS national programme for pregnant women, is one of the mHealth services that has been implemented successfully in SA. However, this national programme did not cater to the specific needs of women living with HIV; therefore, not much is known about the acceptability of mHealth among women living with HIV in SA.^22^ A randomised controlled trial conducted in Uganda found that the text messaging of adolescents and young adults was ineffective in improving ART adherence, despite a year of study.^23^ This study highlights the importance of studying new interventions in different communities.

We evaluated an SMS-based mHealth intervention’s acceptability and explored its impact on follow-up EID uptake in Durban, SA.

## Methods

This study was a descriptive cohort study that also included telephonic interviews with some (*n* = 66, 26%) of the participants. Two hundred fifty-one mothers living with HIV and their infants were enrolled from King Edward VIII Hospital (KEH), an 852-bed hospital serving an urban and peri-urban community in the eThekwini district of KwaZulu-Natal, SA. The hospital has approximately 7000 deliveries per year, with 40% of infants exposed to HIV. These mother–infant pairs (MIPs) were recruited from the postnatal and neonatal wards via consecutive sampling (Monday through Friday) between December 2018 and October 2019.

Recruitment was integrated with the routine postnatal well-baby examination conducted before discharge. For HIV-exposed infants, this included starting appropriate ARV infant prophylaxis and doing a birth HIV PCR test, together with appropriate counselling. The HIV-exposed infants were defined as either low risk (LR) if the mother was on ART with an HIV VL of < 1000 copies/mL in the last three months or high risk (HR) if the mother had no HIV VL available or an HIV VL of > 1000 copies/mL. The mother was approached by a research assistant, who provided information about the study and obtained informed consent. A rotation of allocated doctors conducted the postnatal examinations on the neonate.

**FIGURE 1:**
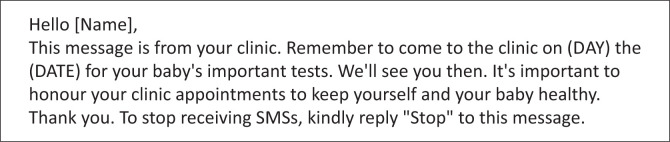
Sample SMS message sent to mothers at 1, 4, 10 and 14 weeks post-delivery.

The enrolment form captured the MIP’s demographic and maternal HIV information. The study participants were informed that they would receive SMS text messages (see Figure 1) that would remind them to attend the clinic for the well-baby and immunisation visits, where the HIV PCR on the infant would be performed as part of the national protocol. The MIP was assigned a patient identity number, and all the details were entered weekly onto a local password-protected Microsoft Excel database accessible only to the study investigators. The cell phone numbers were stored on a secure server managed by Sawubona Health (Malden, MA, United States [US]). Personalised SMS reminders were automatically sent to all recruited participants at weeks 1, 4, 10 and 14 post-delivery via BulkSMS (Celerity Systems Ltd, SA). The wording of the text messages was piloted and developed by the study team using a focus group of postnatal mothers from KEH prior to starting the study. The messages were sent in the participants’ home languages. Participants could freely opt out of the SMS programme at any point by sending a no-cost response to any one of the SMSs that they received.

A telephonic interview (see Appendix 1) was conducted between 14 and 18 weeks of life to verify maternal and infant demographic details; capture information on clinic immunisation attendance, adherence to infant prophylaxis and HIV PCR uptake; and evaluate the intervention’s acceptability. Adherence to infant prophylaxis was based on self-reporting by the mothers. All participants were called for a telephonic interview; however, not all were reached, as some numbers were no longer in use, and some participants did not take the calls. The telephonic interviews, conducted in the patients’ preferred language, were recorded and later translated and transcribed into English. For thematic analysis, interview transcripts were uploaded onto Dedoose software version 8.0.42, 2018 (SocioCultural Research Consultants, LLC, Los Angeles, California, US; www.dedoose.com). We used an inductive content analysis approach that was derived from reviewing, coding and interpreting the data.

A codebook was developed using operational definitions and selected illustrative quotes. The codebook was then refined using an iterative process. Following the completion of the codebook, the remainder of the transcripts were coded using Dedoose software. Categories were developed using coded data to assess the intervention and facilitators’ acceptability, feasibility and barriers to successful preparation and medication administration. The categories were further organised, final definitions created and evidence provided using illustrative quotes from the research participants.

All HIV DNA PCRs performed at birth (within the first three days of delivery) and follow-up EID (done between 10 and 14 weeks of life) were actively checked through the National Health Laboratory Services online portal, and positive results were communicated to the clinical team at KEH for immediate action as per the local protocol. The follow-up EID rates obtained from this study were compared to the follow-up EID rate of 69.3% obtained in 2019 from 53 863 HIV PCR tests that were performed among HIV-exposed infants in the eThekwini district at approximately 10 weeks of life.^24^

Categorical variables were described and summarised by percentages and continuous variables by medians and interquartile ranges (IQR). Univariate analyses comparing the uptake of follow-up EID testing and the characteristics associated with HR and LR mothers and their infants were performed using Student’s *t*-test or the Mantel–Haenszel χ^2^ test for categorical variables. For each univariate analysis, all available case information was used. *P*-values of less than 0.05 were considered significant. All two-way interactions were evaluated using Stata/IC version 15 (College Station, Texas, US).

### Ethical considerations

The research was approved by the Biomedical Research Ethics Committee (BREC) of the University of KwaZulu-Natal (BE4/18), King Edward VIII Hospital (KE 2/7/1/[63/2018]) and the KwaZulu-Natal Department of Health (KZ 201810_013).

## Results

Two hundred and fifty-one MIPs were recruited between December 2018 and October 2019. The median age of the mothers was 30 years (IQR: 9), with predominantly black Africans (98.8%) and multiparous women (73%). The average age for HR mothers was 28 years (IQR: 23–33) and for LR mothers, 31 years (IQR: 27–35).

Out of the 251 MIPs, 178 (70.8%) infants were classified as LR and 73 (29.2%) as HR. Among the HR MIPs, 53 (72.6%) had an undocumented HIV VL and 20 (27.4%) had an HIV VL of > 1000 copies/mL at delivery. Mothers of HR infants were significantly younger than those of LR infants (*P* = 0.003). Late booking (first prenatal care visit later than 20 weeks) was more common in mothers of HR infants than in the LR group, although not statistically significant (*P* = 0.06).

The CD4 counts were available for 178 (70.9%) of the mothers. The percentages of those with CD4 counts of < 500 (36.2%) and > 500 (34.6%) were similar among HR and LR mothers.

The infant cohort (Table 1) comprised mostly term infants (70.1%) that weighed between 2500 g and 3999 g (73%). The infants of HR mothers were significantly more likely to have low birthweight (LBW) (< 2500 g) than those of LR mothers (9.59% vs 7.87%, *P* = 0.029). The mean birthweight of infants of HR mothers was 3050 g and of LR mothers was 3146 g. Infants in this cohort were more likely to be delivered by caesarean section (59.7%) than normal vertex delivery (40.2%), as this is a referral unit that accepts complicated deliveries, although the LR infants were more likely to be born via caesarean section than the HR infants (*P* = 0.04).

**TABLE 1:** Description of maternal and infant characteristics.

Characteristic	High risk (*n* = 73)		Low risk (*n* = 178)		Total (*n* = 251)	*P*
	*n*	%	*n*	%		
**Maternal**						
Age group						
< 25	21	29.60	25	14.10	46	0.003
25–35	40	56.30	113	63.30	155	
36–44	10	14.10	40	22.60	50	
Parity						
Nulliparous	19	26.76	48	27.00	67	0.9
Multiparous	52	73.24	130	73.03	182	
Booking status						
Early (< 20 weeks)	28	38.36	85	47.70	113	0.06
Late/unbooked	31	42.47	77	43.30	108	
Unrecorded	14	21.90	16	9.00	32	
CD4 count						
< 100	1	1.37	2	1.10	3	0.12
100–250	4	5.48	13	7.30	17	
250–500	19	26.03	52	29.21	71	
> 500	14	19.20	73	41.01	87	
Unknown CD4	35	47.90	38	21.35	73	
Infant						
Birthweight						
< 2500 g	7	9.59	14	7.8	21	0.029
2500 g – 3999 g	47	64.38	137	76.97	184	
≥ 4000 g	2	2.74	10	5.62	12	
Unrecorded	17	23.29	17	9.55	34	
Gestational age						
Preterm (< 37/40 weeks)	19	26.03	33	18.54	52	0.4
Term	48	66.00	128	71.91	176	
Unrecorded	6	8.22	17	9.55	34	
Mode of delivery						
Caesarean section	36	51.43	114	64.04	150	0.04
Natural vaginal delivery	20	28.57	48	26.97	68	
Unrecorded	16	20.00	16	8.99	30	

Birth HIV PCRs (Table 2) were performed for most of the infants, 238 (94.8%), with only 1 (0.4%) positive and 1 (0.4%) indeterminate. Infant prophylaxis was correctly prescribed to most infants (247 of 251, 98.4%). However, 3 (1.1%) HR infants were incorrectly prescribed LR-PNP, and 1 (1.1%) LR infant was incorrectly prescribed HR-PNP. The birth HIV PCRs of 11 infants (7 HR and 4 LR) were not found on the online system and were likely not done.

**TABLE 2:** Birth and 10-week polymerase chain reaction.

HIV PCR	High risk (*n* = 73)		Low risk (*n* = 178)		Total (*n* = 251)	*P*
	*n*	%	*n*	%		
**Birth PCR**						
Negative	64	87.67	174	97.50	238	0.002
Indeterminate	1	1.40	0	0.00	1	0.3
Positive	1	1.40	0	0.00	1	0.3
Not obtained	7	9.59	4	2.25	11	0.02
**10-week PCR**						
Negative	48	65.80	136	76.40	184	0.08
Died	1	1.40	0	0.00	1	0.29
Not done	24	32.88	42	24.00	66	0.16

PCR, polymerase chain reaction.

The infant with a positive birth HIV PCR test result was traced as per the hospital tracing protocol and returned to the local clinic on day nine when a confirmatory HIV PCR test was performed. Unfortunately, ART was not started as per current guidelines. The confirmatory HIV PCR test was positive, but the patient was lost to follow-up and was admitted two months later with severe pneumonia and subsequently died. The delay in ART initiation was possibly the result of maternal refusal or of health system failure. Linking these patients with local community caregivers, a system already in place in KwaZulu-Natal, could have been useful to trace this MIP. Perhaps a two-way SMS system could also be useful to trace such patients lost to follow-up, where they could respond with reasons for not coming in for HIV PCR results.

The HIV PCR test rate at 10 weeks after the mHealth intervention was found to be 73.3%, 48 (62.7%) HR and 136 (73.9%) LR infants (Table 3). This rate is higher than the standard of care of 69.3% in the eThekwini District in 2019; however, we cannot conclude from this data set that the higher rate in this cohort was the result of the mHealth intervention, as there was no comparison group. In addition, the increase in the HIV PCR test rate was not statistically significant, 73.3% versus 69.3% (*P* = 0.08).

**TABLE 3:** Maternal and infant risk factors for not obtaining a 10-week polymerase chain reaction.

Risk factors	10-week PCR not obtained (*n* = 67)		10-week PCR obtained (*n* = 184)		Total (*n* = 251)	*P*
	*n*	%	*n*	%		
**Risk**						
Low	42	62.69	136	73.91	178	0.08
High	25	37.31	48	26.09	73	
**Maternal**						
Age group						
< 25	13	20.00	33	18.03	46	0.9
25–35	40	61.54	112	61.20	152	
36–44	12	18.46	38	20.77	50	
Parity						
Nulliparous	15	23.08	52	28.26	67	0.42
Multiparous	50	76.92	132	71.74	182	
Prenatal care						
Early (< 20 weeks)	29	47.54	84	52.50	113	0.51
Late/none	32	52.46	76	47.50	108	
CD4 count						
< 500	17	42.50	74	53.62	91	0.22
> 500	23	57.50	64	46.38	87	
Infant						
Birthweight						
< 2500 g	6	10.34	15	9.43	21	0.88
2500 g – 3999 g	48	82.76	136	85.52	184	
≥ 4000 g	4	6.90	8	5.03	12	
Gestational age						
Preterm (< 37 weeks)	7	11.86	45	26.63	52	0.02
Term	52	88.14	124	73.73	176	

PCR, polymerase chain reaction.

The maternal and infant risk factors that influence a mother’s likelihood to return for a 10-week HIV DNA PCR test are detailed in Table 3. Low-risk mothers were more likely to return than HR mothers (*P* = 0.08). Maternal age, parity, prenatal care, CD4+ T-cell count and infant birthweight were found not to influence the return for 10-week HIV DNA PCR test. Mothers of preterm infants were more than twice as likely to return compared to those of term infants.

Telephonic interviews were conducted in a subset of 66 (26%) participants with no significant differences in age and parity (Table 4) compared to the overall cohort. Unfortunately, the subset is a small percentage of the study participants because of the difficulty reaching many participants telephonically.

**TABLE 4:** Interviewed versus non-interviewed women.

Maternal parameters	Interviewed (*n* = 66)				Not interviewed (*n* = 185)				*P*
	High risk (*n* = 19)		Low Risk (*n* = 47)		High risk (*n* = 54)		Low risk (*n* = 131)		
	*n*	%	*n*	%	*n*	%	*n*	%	
**Age (in years)**									
< 25	7	36.8	7	14.9	14	26.9	18	13.8	0.74†
26–35	10	52.6	34	72.3	30	57.7	78	60.0	
> 36	2	10.5	6	12.8	8	15.4	34	26.2	0.16‡
**Parity**									
Nulliparous	7	36.8	15	31.9	12	23.1	33	25.2	0.24§
Multiparous	12	63.2	32	68.1	40	76.9	98	74.8	0.37¶

HR, high risk; LR, low risk.

† , HR interviewed versus HR not interviewed;

‡ , LR interviewed versus LR not interviewed;

§ , HR interviewed versus HR not interviewed;

¶ , LR interviewed versus LR not interviewed.

Out of the 66 interviews, 53 (80%) considered their clinic to be ‘close by’ (took one local taxi or walking distance), and 59 (90%) reported having attended all the clinic dates. Only 42 provided complete answers to questions about infant prophylaxis. Despite routine postnatal counselling of the mothers regarding infant prophylaxis, only 14 of 42 (33.3%) of the participants knew the infant prophylaxis medication by name, the total duration of administration and when to administer it. The remainder of the participants, 28 of 42 (66.6%), had incomplete knowledge about the infant prophylaxis medication names, duration and administration. Of note, the majority (78%) of the LR participants had complete knowledge of infant prophylaxis (i.e. dose, name of the treatment used and duration) compared to 22% of HR participants.

This poor maternal knowledge of ART translated to poor adherence to infant prophylaxis. Adherence was overall poor among all the mothers interviewed. However, it was even worse among the HR versus LR mothers, but the statistical significance was not calculated, as the numbers were too small and the aim of the telephonic interviews was to collect qualitative data. Out of 42 mothers (5 HR and 37 LR) who answered questions on adherence, none of the five HR mothers gave ART correctly, while 12 of LR mothers gave it correctly.

### High dependence on clinic staff on when to stop infant prophylaxis

We found that the mothers depended heavily on the nursing staff at the busy local clinics to tell them when to stop using infant prophylaxis, and they were mostly unaware of the expected duration for themselves.

When asked about the duration of prophylaxis, this is what some moms had to say:

‘They just said I must give him until it is finished.’ (Mother 12, KH00419, 29-year-old female)

‘She drank it and got immunised at 6 weeks and carried on with it, and we took the other bloods, and they said she must stop taking it.’ (Mother 18, KH01619, 34-year-old female)

### Poor knowledge of antiretroviral treatment names

The mothers generally did not know the names of the ARV drugs that their infants were using. When asked the names, most mothers would describe the colour of the medication only. When given the names of the commonly used medication for infant prophylaxis, most could still not identify which the child was taking. Some of them also confused the HIV prophylaxis with the prophylaxis used for opportunistic infections. This is what some of the moms said when asked about the infant prophylaxis names:

‘She used to take the one in a white bottle, clear in colour, I don’t remember the name.’ (Mother 69, KH01519, 23-year-old female)

‘She was taking the white one.’ (Mother 34, KL02419, 30-year-old female)

‘She is taking the medication for prevention, the white one.’ (Mother 56, KL09419, 34-year-old female)

### Importance of SMS reminders

The majority, 98% (49/50), of the mothers who responded to mHealth questions found the mHealth intervention helpful as appointment reminders, and 73.9% (34/46) were comfortable receiving the SMS messages, as indicated by the following quotes:

‘It’s very important, for instance, if I am on night shift and sleeping during the day when I wake up, I see the SMS and remember.’ (Mother 2, KL02019, 25-year-old female)

‘They are important because I sometimes wouldn’t check the baby’s card, and the SMS will remind me, and then I would quickly check the card.’ (Mother 18, KH01619, 34-year-old female)

### Sharing mobile devices and confidentiality

Sharing of mobile devices was common (30/37); however, most participants expressed that they did not have a problem with a family member reading the SMS sent, as expressed in the following quotes:

‘They use it here at home, but they know my status.’ (Mother 15, KH01119, 35-year-old female)

‘The only person who can see them is my baby’s dad because he knows our situation.’ (Mother 24, KL00519, 29-year-old female)

Accidental HIV disclosure was a concern to three out of 30 of the participants, even though the SMS sent was non-disclosing, and some of the participants emphasised the need to keep the wording of the messages discrete. One of the moms said:

‘It is important, but it must not include full details and what it’s about but just generally remind a person to go to the clinic.’ (Mother 22, KL00219, 34-year-old female)

## Discussion

In this study evaluating an SMS-based mHealth intervention to enhance EID uptake and assess maternal knowledge and adherence to infant ARV prophylaxis among the HR and LR MIPs, the intervention was found to be useful by the interviewed participants, although we could not prove that it promotes adherence. This finding adds to the literature confirming the acceptability of mHealth interventions in various populations.^16,25,26^

Increased uptake in follow-up EID testing was noted compared to the testing rate reported by the Department of Health in the district during the same period; however, the increase was not statistically significant. The follow-up EID testing rates in the district in 2019 were higher than those reported previously in SA. In a study looking at the impact of national guidelines shifting from 6-week HIV PCR to birth HIV PCR in the whole province of KwaZulu-Natal in 2015, Smith et al.^3^ reported that only 14.7% of children with an initial negative birth PCR had a follow-up PCR. While the percentage of HIV PCR follow-up testing was estimated to be only 49% in a study done in Cape Town in 2016, we believe that the SMS reminders can play a role in improving adherence to follow-up EID testing of the PMTCT cascade.^11^ The district’s quality improvement initiatives, duplicate testing and testing of infants coming into the district may account for the higher testing rates. The addition of the mHealth intervention and improved service delivery likely had a smaller than expected benefit that this study was not powered to detect.

Clinic attendance for routine immunisation, which was high among the interviewed participants in this study, does not translate to high follow-up EID uptake. This emphasises the need to integrate PMTCT services into routine immunisation services instead of it being run separately. Other studies in SSA have identified that poor maternal knowledge about vertical transmission of HIV, fear of stigmatisation and lack of privacy at follow-up clinics contribute to poor EID uptake.^12,13^ The findings of poor maternal knowledge on PMTCT may have contributed to poor adherence, and causality could not be proven. The addition of maternal education on PMTCT and EID to mHealth interventions should be further evaluated. Several studies have shown that high knowledge levels among women living with HIV on ART are associated with higher ART adherence.^27,28^ The participants in this study were not interviewed about their ideas of stigmatisation and lack of privacy, which would be an important area to explore in future studies.

Although the health professionals followed the PMTCT guidelines well, for the most part, we found that there is still room for improvement, such as in prescribing the correct PMTCT and in the case of the infant who tested positive but was not initiated onto ART on the day the mother and infant reported to the clinic to obtain the results. A strong link with the community caregiver and social workers to trace MIPs lost to follow-up would also be beneficial. Continued quality improvement programmes and support for overwhelmed clinic health workers in national PMTCT protocols is still needed. A study by Doherty et al. found a lack of ownership of the PMTCT programme among nurses, unclear roles and responsibilities, lack of knowledge of the protocol, poor recording systems and poor continuity of care as factors hindering PMTCT in a study done at a district in KwaZulu-Natal. In addition, the study found that after implementing a participatory approach, the staff and managers identified the delivery of effective PMTCT programmes in their districts and found solutions, together with regular, data-driven, facility-level support.^29^

When comparing HR and LR MIPs, we found younger maternal age, late antenatal booking, poor maternal knowledge of infant prophylaxis and an increased frequency of LBW babies. In the national PMTCT protocol, the HR mothers have been described as at HR of transmitting HIV solely based on their VL or absence thereof within 3 months of giving birth. These findings indicate that these mothers are not only at HR of transmitting HIV, but they and their infants are also at increased risk of several other problems. Late booking alone is associated with poor maternal health, increased child mortality rates and increased risk of transmitting certain infections such as syphilis and malaria, all factors that the Millennium Developmental Goals aimed to combat.^30^ Literature has also shown that small-for-gestational-age and LBW infants are at significantly increased risk of mortality and morbidity compared with appropriate-for-gestational-age infants.^31^ These findings mean that our health system should have more stringent criteria for following these high-risk MIPs to ensure a holistic care approach.

### Strength and limitations

Limitations noted in this study include self-reporting by the mother to assess adherence to infant prophylaxis. Although this often introduces recall bias, several studies have promoted self-reporting to estimate ART adherence in developing settings where objective measures are often expensive or impractical.^32,33^ Adherence to maternal ART was not assessed.

## Conclusion

mHealth via SMS reminders is a helpful tool in promoting immunisation clinic visits; however, we could not prove that it is associated with increased EID uptake. Maternal knowledge of PMTCT and EID is still key, and future mHealth studies should explore the use of these interventions to increase knowledge and empower women living with HIV. A holistic approach to the care of HR mothers should address the factors that may impact maternal and infant health.

## APPENDIX 1

### Telephone survey script

Hello, my name is______________________I am with the SMS/prophylaxis research team here at King Edward Hospital. I am calling because you signed up for our SMS reminder programme. Does this sound familiar to you?

(If **No:** Apologise for having the wrong number and kindly end the call.)

(If **YES … continue**)

We are calling up participants of the programme to find out about their attitudes and experiences using the Sawubona SMS reminder programme and also to find out about the medication you gave to your baby. If you have a few minutes, I’d like to ask you a few questions that will take about 10 min. It is important that you tell me exactly what is in your mind rather than something you think I want to hear. This phone call is not being recorded and what you say here will not be shared with anyone at the hospital or the clinics you attend. The information you provide will be kept confidential. Do you have a few minutes to chat?

(If **No** … try to reschedule the call for a later time)

(If **Yes … continue**)

1. Recruitment/Enrolment

**Table:** During the enrolment phase, when you signed up for the programme, did you experience any of the following?

Reaction	Response		Comments
	Yes (1)	No (2)	
1. Trouble understanding purpose of research project and your role in it			
2. Discomfort giving name to research staff member			
3. Discomfort giving personal phone number to research staff member			
4. Unease with phone number confirmation procedure (i.e. replying with ‘yes’ or ‘ yebo’ to initial SMS)			
5. Worry that collected personal information could be lost			
6. Worry that collected personal information could be shared with third parties, for example telemarketers			
7. Other concerns			

2. Acceptability

**Table:** In the course of the study, when you were receiving SMS reminders for your appointments, did you experience any of the following?

Reaction	Response		Comments
	Yes (1)	No (2)	
1. Problems (not) receiving SMS reminders at the right (wrong) time			
2. Problems with inaccurate/inappropriate SMS content (e.g. names mix up)			
3. Problems understanding the SMS content			
4. Fear/worry that others might see the SMS reminder on your phone			
5. Feel bothered by the SMS (e.g. felt like it was spam)			
6. Other people asking you about the SMS			
7. Trouble opting out of the programme (for those who have opted out)			
8. Any other adverse experiences			

3. Perceived usefulness

**Table:** What effect did the reminders have on your remembering of your appointments? How?

Effect	Comment
Positive	
Neutral/no effect	
Negative	

**Table:** What effect did the reminders have on your attendance of your appointments? How?

Effect	Comment
Positive	
Neutral/no effect	
Negative	

**Table:** Would you recommend that other mothers enrol for the programme? Why?

Response	Comment
Yes	
No	

**Table:** Overall how much did you like using/being a part of the Sawubona SMS reminder programme?

Effect	Comment
Liked it	
Neutral	
Did not like it	

Using a 10-point scale, how important was this programme to your health and that of your baby in the time that you were enrolled? (1 = least important; 10 = very important)

**Next, I would like to ask a few questions about the health of your baby and attendance at your local health clinic.**

1. Please confirm your baby’s sex
  - Male
  - Female
2. Please confirm your baby’s name, surname and date of birth
3. Which of the following medications was baby given at the hospital?
  - NVP
  - AZT
  - Both
  - Other
4. How many doses of those medications (AZT, NVP, other) did your child receive on most days?
  Select from the following:
  - None
  - Once a day
  - Other
5. At how many weeks of life did you last attend the clinic?
6. Did your baby receive immunisations at six weeks?
7. Did your baby receive immunisations at 10 weeks?
8. For how many weeks have you given your baby the medicine?
  Select from following:
  - Less than 5 weeks
  - 6 weeks
  - 6–10 weeks
  - 10 weeks

### Conclusion

What are your thoughts on this survey? Are there ways you think we can use to improve it?

Do you have any questions for me about the programme or anything we’ve talked about today?

Thank you very much for your time. Your responses have been very helpful. Have a nice day.

[*End*]
